# Taurine attenuates oxidative stress and alleviates cardiac failure in type I diabetic rats

**DOI:** 10.3325/cmj.2013.54.171

**Published:** 2013-04

**Authors:** Guo-guang Wang, Wei Li, Xiao-hua Lu, Xue Zhao, Lei Xu

**Affiliations:** 1 Department of Pathophysiology, Wannan Medical College, Wuhu, China; 2 Department of Biochemistry, Wannan Medical College, Wuhu, China

## Abstract

**Aim:**

To investigate cardioprotective effect of taurine in diabetic rats.

**Methods:**

Male Sprague-Dawley rats were assigned randomly into four groups of 15 rats: control group, control + taurine group, streptozotocin (STZ) group, and STZ + taurine group. Rats in STZ and STZ+ taurine groups were treated by a single injection of STZ (70 mg kg^-1^, intraperitoneally) dissolved in 0.01 M citrate buffer (pH 4.5) for induction of diabetes, and rats in control and control + taurine groups were treated with the same volume citrate buffer. Taurine was orally administered to rats in control + taurine and STZ + taurine groups daily for 8 weeks. Rats were examined for diabetic cardiomyopathy by left ventricular (LV) hemodynamic analysis. Myocardial oxidative stress was assessed by measuring the activity of superoxide dismutase (SOD) and the level of malondialdehyde (MDA). Myocardial protein kinase B (Akt/PKB) phosphorylation and heme oxygenase-1 (HO-1) protein levels were measured by Western blot in all rats at the end of the study.

**Results:**

In untreated diabetic rats, LV systolic pressure, rate of pressure rise, and rate of pressure fall were decreased, while LV end-diastolic pressure was increased, indicating reduced LV contractility and slowing of LV relaxation. The levels of Akt/PKB phosphorylation and SOD activity were decreased and HO-1 protein expression and MDA content increased. Taurine treatment significantly improved LV systolic and diastolic function, and there were persistent increases in activities of Akt/PKB and SOD, and the level of HO-1 protein.

**Conclusion:**

Taurine treatment ameliorates myocardial function and heart oxidant status, while increasing myocardial Akt/PKB phosphorylation, and HO-1 levels have beneficial effects on diabetic cardiomyopathy.

Diabetes mellitus is a serious medical problem and has become a significant health concern. Diabetic patients can develop a specific cardiomyopathy called diabetic cardiomyopathy (DCM) (1), which is an important contributing factor to heart failure in diabetic patients independent of atherosclerosis, hypertension, and other complications (2,3). Mechanisms responsible for DCM are still poorly understood, but direct toxic effect of hyperglycemia on cardiomyocytes and oxidative stress are hypothesized to play an important role in the pathogenesis of diabetes and its late complications (4). Hyperglycemia can lead to oxidative stress by a wide range of mechanisms, including glucose auto-oxidation, increased production of advanced glycosylation end products, and activation of the polyol and hexosamine pathways (5,6). Oxidative stress is thought to contribute to the initiation and progression of cardiac dysfunction and remodeling of the extracellular matrix in the heart (7-9).

Heme oxygenase-1 (HO-1) is a ubiquitously expressed stress inducible enzyme that catabolizes heme into bilirubin, carbon monoxide (CO), and iron (10). The by-products of heme catabolism exert pleiotropic cytoprotective effects in the heart. Bilirubin is a powerful antioxidant (11), and CO exerts vasodilatory, anti-inflammatory, and anti-proliferative effects (12). Enhanced HO-1 expression enhances the concentration of p-AKT (13). Moreover, a large body of evidence has shown that activation of AMP-activated protein kinase (AMPK) and phosphorylated protein kinase B (p-Akt/PKB) increases the phosphorylation of a number of target molecules, resulting in increased glucose transport, fatty acid oxidation (14), and protection against oxidative stress (15-17).

Taurine (2-aminoethanesulphonic acid) is the major intracellular free β-amino acid, which is one of the most abundant free amino acids present in mammalian tissues and blood cells. It is involved in many important biological and physiological functions, such as anti-oxidation, osmoregulation, membrane stabilization, and neurotransmission (18). Taurine is a protective agent against oxidative stress-induced pathologies such as atherosclerosis (19), diabetic complications (18), and gastrointestinal (20) damage. It also has anti-apoptotic properties and inhibits oxidative stress-induced apoptosis in several cells, such as hepatocytes (20).

The aim of this study was to examine whether taurine increases HO-1 expression and ameliorates cardiac failure in type I diabetic cardiomyopathy. We examined left ventricular (LV) hemodynamic function, evaluated myocardial oxidative stress, and myocardial levels of HO-1, connective tissue growth factor (CTGF), and p-AKT in the heart.

## Methods

### Experimental animals and drug treatment

All animal procedures and experiments were conducted in accordance with the official recommendations of the Chinese Community Guidelines. Male Sprague-Dawley rats, weighting 180-220 g, were provided by the Experimental Animal Center in Wannan Medical College and housed in a standard animal facility under controlled environmental conditions at room temperature, 22 ± 2°C and 12-hour light-dark cycle. Sixty rats were kept for 1 week on balanced ration with water and food *ad libitum* for acclimatization. The animals were housed in stainless steel cages with sawdust bedding. Diabetes was induced by injecting a single dose of streptozotocin (STZ) (70 mgkg^-1^, intraperitoneally, dissolved in ice-cold sodium citrate buffer, 0.1M, pH 4.5), as previously described (21). Thirty age-matched control rats were treated with the same volume citrate buffer. Diabetes was confirmed 48 hours after STZ injection by measuring the glucose concentrations of peripheral blood obtained from the tail vein (One Touch SureStep Meter, LifeScan, Milpitas, CA, USA). Diabetes was diagnosed by a sustained glucose concentration >15 mmol/L. Taurine (100 mg kg^-1^ per day) was administered to the rats in the control + taurine and STZ + taurine group, and animals had free access to water after diabetes induction.

### Cardiac function

After eight weeks of drug dosing, rats were anesthetized with sodium pentobarbital (50 mg/kg i.p.). The right carotid artery was cannulated with a Millar miniature catheter and advanced into the aorta to record arterial pressure. The aortic catheter was then advanced into the left ventricle for recording of left ventricular systolic pressure (LVSP), left ventricular developed pressure (LVDP), left ventricular end diastolic pressure (LVEDP), maximum rate of rise/fall of left ventricle pressure (±dP/dtmax). All pressure data were recorded with MedLab data acquisition system (Nanjing MedEase Co., Nanjing, China) and fasting blood samples and hearts were collected and investigated on the same day.

### Biochemical markers of myocardial injury in serum

Estimation of total cholesterol (TC), triacylglycerol (TG), low density lipoprotein (LDL), high density lipoproteins (HDL), creatine kinase (CK), and lactate dehydrogenase (LDH) in the serum was done using appropriate kits on semi auto analyzer by well standardized methods (22).

### Determination of oxidative and anti-oxidative status

Heart homogenate (10%, w/v) was prepared with 0.1M PBS and centrifuged at 1200 g for 10 minutes. The supernatant was used to determine SOD activity and MDA level with commercially available kits (Nanjing Jiancheng Bioengineering Institute).

### Western blotting analysis

Heart samples (0.2 g) were lysed and homogenized in 2 mL of lysis buffer (10 mM Tris-buffered saline, 1 mM EDTA, 1 mM EGTA, 2 mM sodium orthovanadate, 0.2 mM PMSF, 2 μg/mL leupeptin, 2 μg/mL aprotinin, and 1% Triton X-100) for 30 minutes on ice and cleared by centrifugation at 13 000 g for 15 minutes at 4°C. Total protein concentration was determined in the supernatant using the Bradford assay (Bio-Rad Laboratories, Hercules, CA, USA). For each lane, equal amounts of protein were mixed with sodium dodecyl sulfate (SDS) sample buffer and boiled for 5 minutes. Samples were separated on a 10% sodium dodecyl sulfatepolyacrylamide gel and transferred to 0.2-μm nitrocellulose membrane. Nitrocellulose blots were blocked by incubation in TBS-T (10 mM Tris-HCl, pH 7.5, 150 mM NaCl, and 0.1% Tween 20) containing 5% non-fat milk for 1 hour at room temperature and incubated with a rabbit polyclonal anti-HO-1, AKT/PKB, phospho-AKT/PKB, CTGF, β-actin antibody (1:500 dilution; Wuhan Boster Biotechnologies, Wuhan, China) overnight at 4°C. After 3 washing steps, a secondary anti-rabbit antibody (1:10 000 dilution; Sigma Chemical Co, St. Louis, MO, USA) was added and incubated for 1 hour. After rinsing with wash buffer for three times, the reaction was visualized by DAB (Bio Basic Inc. Markham Ontario, Canada). The relative amounts of the bands were quantified by densitometry using image software.

### Statistical analysis

Results are expressed as mean ± standard error of the mean of the values. Statistical significance for all variables was determined by analysis of variance (ANOVA) followed by a Tukey post hoc analysis by using SPSS, version 16.0 (SPSS Inc., Chicago, IL, USA). *P*-values of <0.05 were considered significant.

## Results

### Change in body weight and heart weight/body weight ratio (HW/BW)

After STZ administration, animals in STZ and STZ+ taurine groups showed similar levels of hyperglycemia. Diabetic rats treated with or without taurine had significantly lower body weight than controls (Table 1). However, STZ-diabetic animals had increased heart/body weight ratio. Taurine treatment prevented body weight loss in diabetic rats compared with diabetic rats untreated with taurine. In addition, diabetic rats had an increased heart weight/body weight ratio, a marker for the development of diabetic cardiomyopathy, and this ratio was significantly decreased by treatment with taurine (Table 1).

**Table 1:** Effect of taurine on blood glucose, body weight (BW), heart weight, and heart weight/body weight (HW/BW) ratio in control rats, control rats treated with taurine, streptozotocin (STZ) diabetic rats, and STZ diabetic rats treated with taurine (100 mg/kg) (n = 8)*

	Control	Control + taurine	STZ	STZ+taurine
Blood glucose (mmol/L)	4.81 ± 0.65	4.73 ± 0.51	24.29 ± 3.09^‡^	22.35 ± 3.07
Body weight (BW) (g)^†^	394.6 ± 15.6	399.4 ± 14.1	169.6 ± 8.7^‡^	275.6 ± 13.9^§^
Heart weight (HW) (g)^†^	1.24 ± 0.06	1.29 ± 0.07	1.05 ± 0.07^‡^	1.10 ± 0.06
HW/BW ratio (mg/g)	3.13 ± 0.19	3.23 ± 0.21	6.22 ± 0.56^‡^	3.99 ± 0.37^§^

*The results are expressed as mean ± standard error of the mean of the values. The number of animals per group was 8 for determination of blood glucose, BW, HW and HW/BW ratio levels.

†Body weight and heart weight were measured after 8 weeks.

‡*P* < 0.05 vs control group.

§*P* < 0.05 vs STZ group.

### Effects of taurine on cardiac function

Left ventricular hemodynamic parameters were measured in taurine-treated and un-treated control and diabetic groups of rats for assessment of ventricular performance. Un-treated diabetic rats showed a lower left LVSP, LVDP, and a higher LVEDP than control rats (Table 2). Also, untreated diabetic rats showed a decreased left ventricular ± dP/d*t*max. Diabetic rats treated with taurine showed increased LVSP, LVDP, and reduced LVEDP, with a higher left ventricular ± dP/d*t*max. Untreated diabetic rats had significantly decreased heart rate (HR). Taurine treatment increased HR (Table 2).

**Table 2:** Homodynamic parameters in control rats, control rats treated with taurine, streptozotocin (STZ) diabetic rats, and STZ diabetic rats treated with taurine (100 mg/kg) (n = 8)*†

	Control	Control + taurine	STZ	STZ+taurine
HR (bpm) ^†^	420 ± 7	427 ± 9	307 ± 7^‡^	361 ± 9^§^
LVSP (mmHg) ^†^	120.5 ± 1.4	124.8 ± 4.7	88.4 ± 2.2^‡^	104.5 ± 4.1^§^
LVEDP (mmHg) ^†^	1.22 ± 0.2	1.15 ± 0.09	4.66 ± 0.86^‡^	3.48 ± 0.56^§^
LVDP (mmHg) ^†^	84 ± 6.5	86 ± 5.5	52.9 ± 3.4^‡^	69 ± 6.2^§^
+d*P*/d*t*max (mmHg/s) ^†^	4285 ± 98	4254 ± 158	2418 ± 78^‡^	3303 ± 81^§^
−d*P*/d*t*max (mmHg/s) ^†^	4180 ± 61	4015 ± 121	2295 ± 85^‡^	3139 ± 98^§^

*Abbreviations: HR – heart rate; LVSP – left ventricular systolic pressure; +dP/dt – rate of pressure rise; −dP/dt – rate of pressure fall; LVEDP – left ventricular end-diastolic pressure; LVDP – left ventricular developed pressure.

†The results are expressed as mean ± standard error of the mean of the values. The number of animals per group was 8 for determination of HR, LVSP, +dP/dt, −dP/dt, LVEDP, and LVDP levels. Homodynamic parameters were measured after 8 weeks.

‡*P* < 0.05 vs control group.

§*P* < 0.05 vs STZ group.

### Effects of taurine on CK and LDH

**Figure 1:**
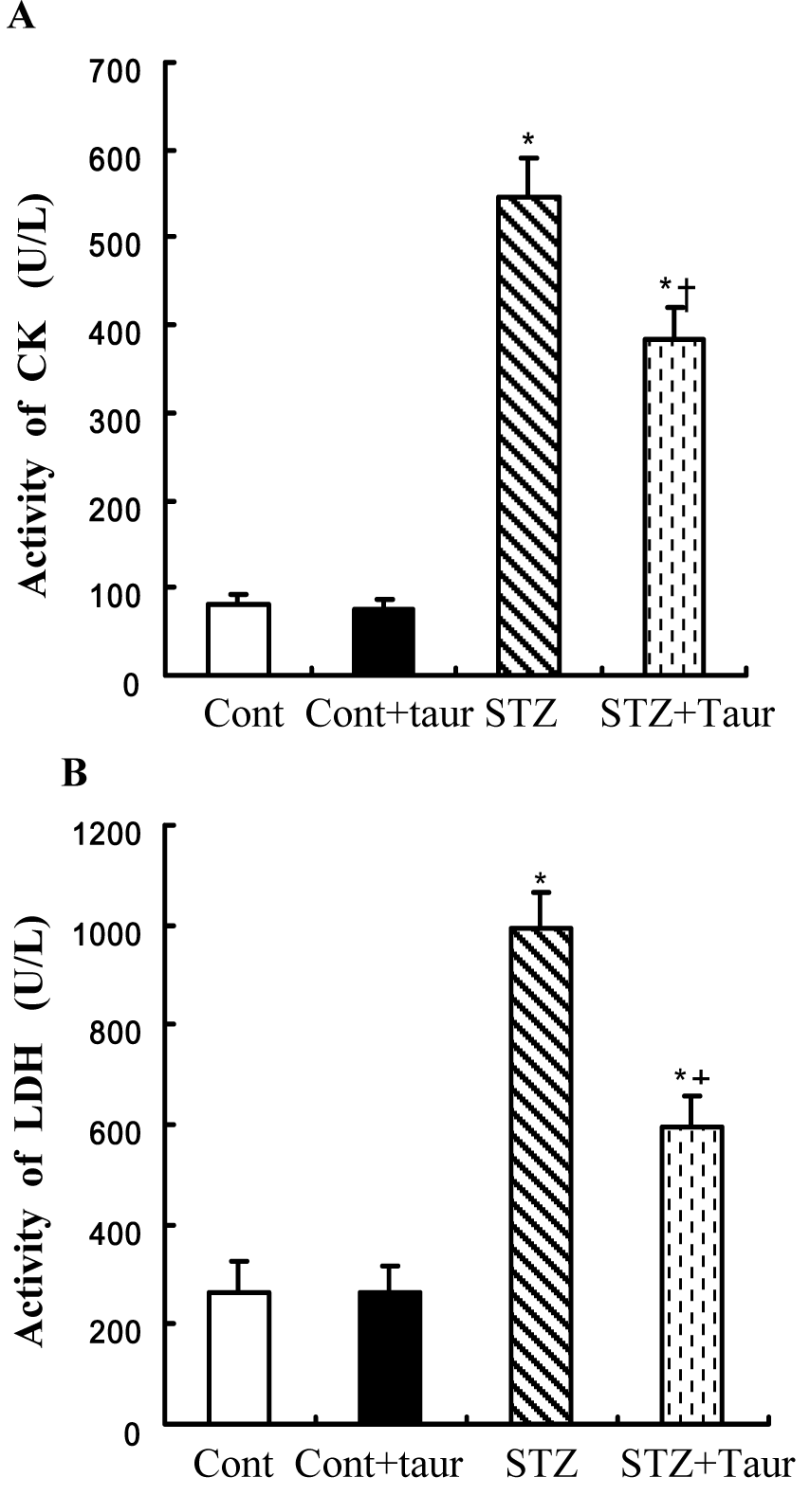
The levels of creatine kinase and lactate dehydrogenase in serum from control and streptozotocin (STZ) diabetic rats treated with or without taurine (100 mg/kg). (**A**) creatine kinase (CK); (**B**) lactate dehydrogenase (LDH). Cont – control rats; cont + taur – control rats with taurine; STZ – diabetic rats; STZ+taur – diabetic rats with taurine. The results are expressed as mean ± standard error of the mean. The number of animals per group was 8 for determination of CK and LDH activities. *P < 0.05 vs control (cont) group, † *P* < 0.05 vs STZ group.

The levels of LDH and CK were significantly higher in untreated diabetic rats than in control rats. However, taurine treatment in diabetic rats markedly reduced the levels of LDH and CK compared with those in untreated diabetic rats (Figure 1).

### Effects of taurine on serum lipids profile

Taurine had a significant effect on lowering triglycerides, cholesterol, and LDL levels and increasing HDL levels in diabetic rats (Table 3). All these levels were completely normalized after taurine treatment, which suggests that taurine is far more effective in maintaining the lipid profile at the levels similar to those of controls in this animal model of diabetes.

**Table 3:** **Effects of taurine on serum lipid profile in control rats, control rats treated with taurine,** streptozotocin (STZ) diabetic rats, and STZ diabetic rats treated with taurine (100mg/kg) (n = 8)*†

	Control	Control + taurine	STZ	STZ+taurine
TC (mmol/L)	1.51 ± 0.33	1.48 ± 0.30	5.04 ± 0.81*	3.05 ± 0.44†
TG (mmol/L)	0.75 ± 0.14	0.72 ± 0.18	2.37 ± 0.38*	1.34 ± 0.31†
LDL (mmol/L)	1.69 ± 0.30	1.66 ± 0.24	6.89 ± 0.92*	3.40 ± 0.57†
HDL (mmol/L)	1.31 ± 0.16	1.33 ± 0.14	0.37 ± 0.09*	0.62 ± 0.12†

*Abbreviations: TC – total cholesterol; TG – triacylglycerol; LDL – low density lipoprotein; HDL – high density lipoproteins.

†The results are expressed as mean ± standard error of the mean of the values. The number of animals per group was 8 for determination of TC, TG, LDL, and HDL levels.

*P < 0.05 vs control group.

†*P* < 0.05 vs STZ group.

### Antioxidant effects of taurine

**Figure 2:**
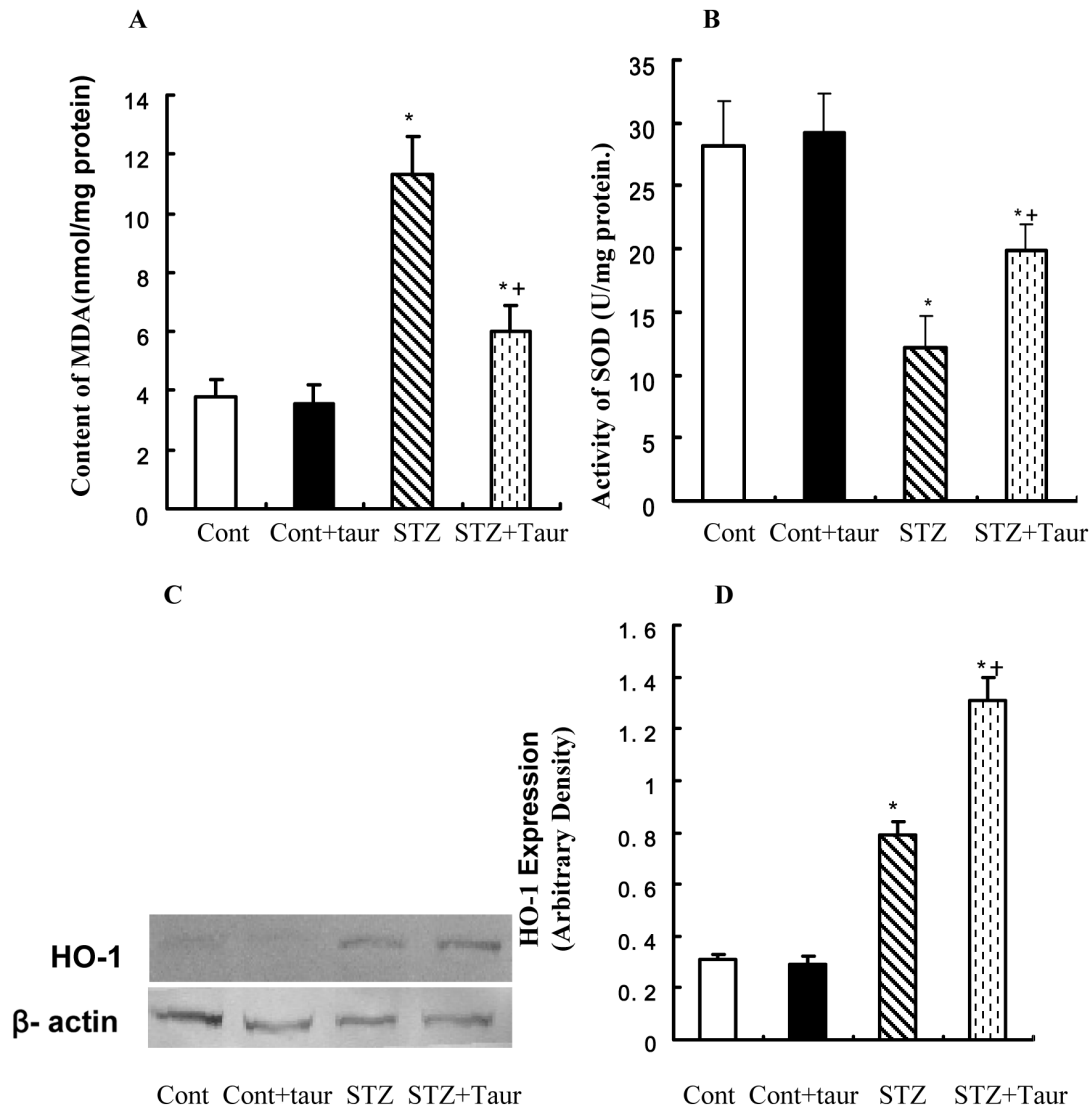
The levels of malondialdehyde and superoxide dismutase in cardiomyocyte from control and streptozotocin (STZ) diabetic rats treated with or without taurine (100 mg/kg). (**A**) malondialdehyde (MDA); (**B**) superoxide dismutase (SOD); (**C**) Representative gel blots of heme oxygenase-1 (HO-1) and β-actin (loading control) using specific antibodies; (**D**) HO-1 expression. Cont – control rats. Cont + taur – control rats with taurine. STZ – diabetic rats. STZ+taur – diabetic rats with taurine. The results are expressed as mean ± standard error of the mean. The number of animals per group was 8 for determination of MDA and HO-1 levels and SOD activity. *P < 0.05 vs control (cont) group, †*P* < 0.05 vs STZ group.

The content of malondialdehyde equivalents in the heart, which reflects oxidant-induced lipid peroxidation reactions, was significantly increased in diabetic animals (Figure 2). Taurine significantly improved the oxidant status, as evident from the reduction of malondialdehyde concentrations. SOD activity was significantly lowered in the hearts of untreated diabetic rats. Treatment with taurine significantly increased the activity of SOD.

Western blot analyses showed an accumulation of HO-1 protein in diabetic rats compared with normal control and normal taurine rats (Figure 2). Taurine treatment led to a strong up-regulation of HO-1 protein compared with untreated rats (Figure 2).

### Expression of CTGF and p-Akt/PKB following taurine treatment

**Figure 3:**
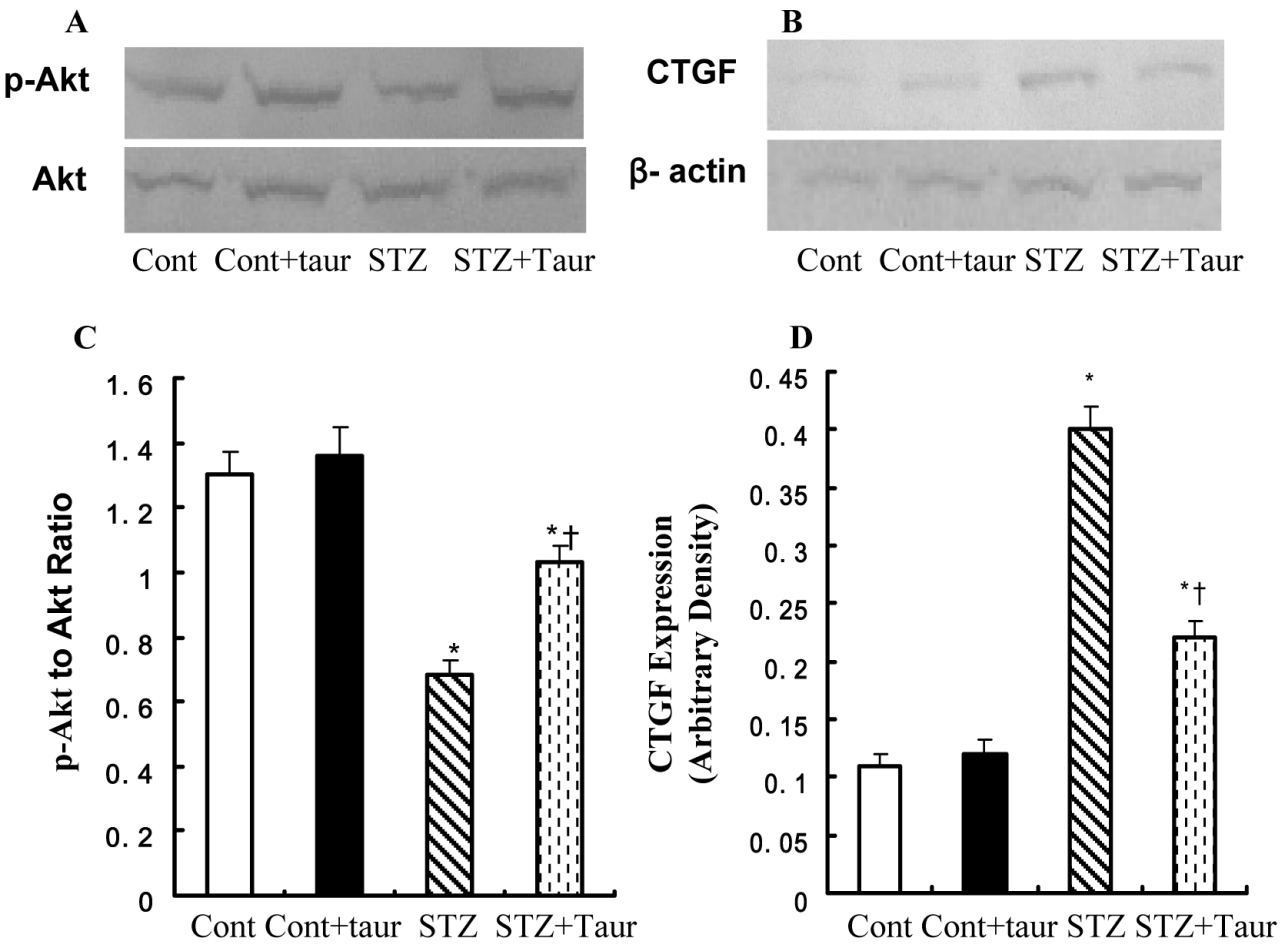
Protein expression of phosphorylated protein kinase B (p-Akt/PKB) and connective tissue growth factor (CTGF) in cardiomyocyte from control and streptozotocin (STZ) diabetic rats treated with or without taurine (100 mg/kg). (**A**) Representative gel blots of p-Akt. (**B**) Representative gel blots of CTGF. (**C**) p-Akt to Akt ratio; and (**D**) CTGF expression. Cont – control rats; Cont + taur – control rats with taurine; STZ – diabetic rats; STZ+taur – diabetic rats with taurine. The results are expressed as mean ± standard error of the mean. The number of animals per group was 6 for determination of p-Akt/PKB and CTGF levels. *P < 0.05 vs control (cont) group, †*P* < 0.05 vs STZ group.

Diabetes led to a reduced expression of p-Akt without affecting the levels of Akt and increased expression of CTGF, while taurine partially restored STZ-induced loss of Akt phosphorylation and reduced the expression of CTGF (Figure 3A and 3B).

## Discussion

This study showed that diabetes enhanced oxidative stress and impaired cardiac function and that treatment with taurine was beneficial to the hearts of diabetic rats as it restrained the progression of metabolic disorders of diabetes. STZ-diabetic rats showed elevated blood glucose levels. Also, there was a notable increase in the HW/BW ratio, which signifies cardiac hypertrophy. Metabolically, the diabetic heart is characterized by diminished glucose utilization and increased fatty acid oxidation, resulting in lipid accumulation in the myocardium (5,6). Taurine treatment showed a distinct positive effect on HW/BW ratio, indicating that taurine was able to prevent cardiac hypertrophy, which usually sets in as a result of diastolic dysfunction secondary to diabetes. Taurine also remarkably improved lipid profile and had a significant effect on lowering triglyceride, cholesterol, and LDL levels and increasing HDL in diabetic rats. Untreated diabetic animals showed significant decreases in LVSP, +dP/dt, and  − dP/ dt, and increases in LVEDP. These hemodynamic alterations demonstrate abnormal left ventricular systolic and diastolic functions, which are the hallmark of diabetic cardiomyopathy. Taurine treatment attenuated these hemodynamic changes.

Increases in levels of circulating cardiac damage markers, such as CK and LDH, represent a powerful and sensitive predictor of increased cardiac complications (23). In the present study, taurine-treated diabetic rats showed a significant improvement in the levels of these markers. This indicates that taurine prevents cardiac damage and has beneficial properties. The decrease in the markers in diabetic rats treated with taurine suggests that taurine reduced the risk of metabolic disorders associated with diabetes.

Increased oxidative stress and altered antioxidant pool have been found in both clinical and experimental type 1 diabetes (24). This was in conjunction with depletion of superoxide scavenger SOD and increase in lipid peroxidation product MDA. Taurine is often referred to as a semi-essential amino acid because it is not used in protein synthesis. It amounts to a large proportion of the total free amino acids in the heart, kidney, and plasma (25) and is vital for normal development. A recent study indicated that taurine was able to ameliorate diabetic cardiomyopathy by down-regulating AT2 receptors (26), and dietary supplementation of taurine attenuated diabetes-induced changes in cardiac contractile function and ultrastructure (27). It was also reported to have beneficial effects in various physiological and pathological conditions by mainly diminishing production of reactive oxygen species (ROS) (18). The present study showed that STZ-induced diabetic condition resulted in an increase in the lipid peroxidation, which is the direct indicator of systemic oxidative stress. Taurine treatment in diabetic rats reduced the formation of lipid peroxides and also restored the levels of SOD in the diabetic hearts. HO-1, the inducible isoform of the HO system, is a rate-limiting enzyme, which converts heme into equimolar amounts of iron, carbon monoxide, and biliverdin. HO-1 is thought to have antioxidant and cytoprotective roles (28). Taurine treatment significantly increased the level myocardial HO-1 in the STZ-diabetic rats. This beneficial effect of taurine could be directly attributable to its antioxidative nature, which is in agreement with previous findings (29). The elevated level of serum lipids in DM causes the risk of coronary heart disease (30), while in some studies, HDL was found to be cardioprotective (31). Serum lipids like TC, TG, and LDL were significantly decreased and HDL was significantly increased in diabetic rats treated with taurine. Taurine is known to have antioxidant properties (18,20) and this may reduce the susceptibility of lipids to oxidation and stabilize the membrane lipids, thereby reducing oxidative stress.

Akt is an important downstream effector of insulin signaling, critical for regulation of cardiac growth and survival (32), while the PI3K/Akt cascade is an important survival pathway in the heart. Akt overexpression leads to increased survival and improved myocardial function (33). Cytoprotective effect of HO-1 is mediated via activation of the PI3K/Akt pathway (34). Insulin has been reported to increase HO-1 mRNA and protein expression in a time- and dose-dependent manner with maximum effects at 3 and 12 hours, respectively. The mechanisms by which insulin promotes HO-1 levels were further analyzed and showed that insulin induced HO-1 mRNA expression through activation of PI3-kinase/Akt pathway (35). HO-1 may be induced by inflammation and/or oxidative stress, which generates transcription factors activated by p-AKT and then increases the level of HO-1 protein (36). Recent evidence suggests that these two enzyme systems interact both at the posttranscriptional and posttranslational levels (37). CTGF has been demonstrated to play an important role in fibrotic response through a transforming growth factor-beta 1 (TGF-β1) -dependent or independent pathway (38). CTGF acts as a cofactor with TGF-β to induce fibroblasts to become myofibroblasts that deposit collagen, ultimately resulting in organ scarring and dysfunction, and in the most severe forms, organ failure and death. Indeed, CTGF levels in tissue, blood, or vitreal fluid have been shown to correlate with the degree and severity of fibrosis in many diseases (39). Other studies showed that CTGF also exhibited prohypertrophic properties on cardiomyocytes (40). Furthermore, CTGF has been proposed as a heart failure biomarker (41) and found to be induced by stress in cultured cardiomyocytes (42). Many studies showed that PKC up-regulated CTGF by inhibiting the PI3K/Akt pathway in the diabetic heart (43). Our study showed that taurine could increase p-Akt expression and antioxidant levels and reduce CTGF expression. This suggests that the protective effect of taurine against DCM takes place at least in part via increasing antioxidant levels and reducing fibrosis.

In summary, the present findings show that treatment with taurine may attenuate the progressive cardiac dysfunction and myocardial oxidative stress in a murine model of diabetic cardiomyopathy. The beneficial effect of taurine is a result of its inhibition of myocardial oxidative stress processes through its potential autoregenerative antioxidant properties. Evidence indicates that any treatment that is able to modulate oxidative stress and enhance antioxidant would help to delay the onset of DCM in diabetes mellitus. Thus, we suggest further exploration of the role of antioxidant therapy given immediately after the diagnosis of type I diabetes mellitus to reduce the risk of future cardiovascular complications.
